# Mediastinal hematoma as an unusual intrathoracic manifestation of Boerhaave Syndrome: A case report

**DOI:** 10.1016/j.ijscr.2024.109366

**Published:** 2024-02-12

**Authors:** Yusuke Nakano, Toru Nakamura, Makoto Tomatsu, Yuichiro Miyaki, Kazufumi Suzuki

**Affiliations:** a Department of Gastrointestinal Surgery, Seirei Hamamatsu General Hospital, 2-12-12 Sumiyoshi, Chuo-ku, Hamamatsu-city, Shizuoka 430-8558, Japan; b Department General Thoracic Surgery, Seirei Hamamatsu General Hospital, 2-12-12 Sumiyoshi, Chuo-ku, Hamamatsu-city, Shizuoka 430-8558, Japan

**Keywords:** Boerhaave Syndrome, Hematoma, Emergency treatment, Case report

## Abstract

**Introduction:**

Boerhaave Syndrome (BS) is rare but life-threatening condition caused by a sudden increase in the intraluminal pressure due to vomiting. We present a case of BS manifesting as a posterior mediastinal hematoma, indicative of a potentially fatal condition.

**Presentation of case:**

A 51-year-old man presented with acute chest pain after vomiting. Enhanced Computed Tomography revealed mediastinal fluid with a left pleural effusion, leading to a diagnosis of BS. Emergency surgery revealed a posterior mediastinal hematoma with active bleeding due to a torn proper esophageal artery. Hemostasis and a wall repair were performed, and the patient was discharged uneventfully.

**Discussion:**

This case highlights two important aspects. Firstly, a spontaneous esophageal perforation can manifest as a mediastinal hematoma due to the subpleural arterial injury, delaying bacterial spillage. While preoperative thoracentesis may not always diagnose BS accurately, bloody thoracic drainage can serve as an alternative diagnostic sign. Secondly, the mediastinal hematoma itself poses a serious risk, as it can lead to a catastrophic outcome even before bacterial contamination occurs, emphasizing the necessity of a timely surgical intervention in BS cases.

**Conclusion:**

BS can manifest as a mediastinal hematoma, and the absence of gastrointestinal content in the thoracic drainage does not rule out the possibility of BS. Prompt surgical intervention remains essential, as a mediastinal hematoma alone can result in a catastrophic outcome. This case highlights the significance of a comprehensive diagnostic assessment for BS.

## Highlights

• Boerhaave Syndrome can manifest as a rare mediastinal hematoma.
• A mediastinal hematoma itself can result in a catastrophic outcome even before the bacterial spillage becomes evident.
• This report highlights the significance of a comprehensive diagnosis and timely surgical intervention for Boerhaave Syndrome.

## 1 Introduction

A spontaneous esophageal perforation, known as Boerhaave Syndrome (BS), is caused by a sudden increase in the intraluminal pressure due to vomiting. The leading etiology is bacterial spillage of the food residue, which can result in life-threatening mediastinitis or empyema [1,2]. It is a rare but often fatal condition, with an annual incidence of only 3.1 cases per 1,000,000 people and a mortality rate exceeding over 30 % [[3], [4], [5], [6]]. The conventional Mackler's triad of chest pain, vomiting, and subcutaneous emphysema, is not so commonly observed, BS often poses a diagnostic challenge [7,8]. Despite successful conservative management as an alternative, prompt surgical intervention has historically been the mainstay of treatment [[9], [10], [11]]. We present a case of BS manifesting as a posterior mediastinal hematoma, indicative of a potentially fatal condition. This work has been reported in line with the SCARE criteria [12].

## 2 Presentation of case

A 51-year-old man with a history of acute pancreatitis and an alcoholic fatty liver presented with acute chest pain and tachypnea following vomiting approximately 9 h after drinking alcohol. The temperature was 36.7 °C, the blood pressure 151/114 mmHg, the pulse 130 beats per minute, the respiratory rate 30 breaths per minute, and the oxygen saturation 99 % under oxygen administration at 5 l/min. The patient was alert and the physical examination revealed a mild left chest wall tenderness without crepitus.

**Fig. 1:**
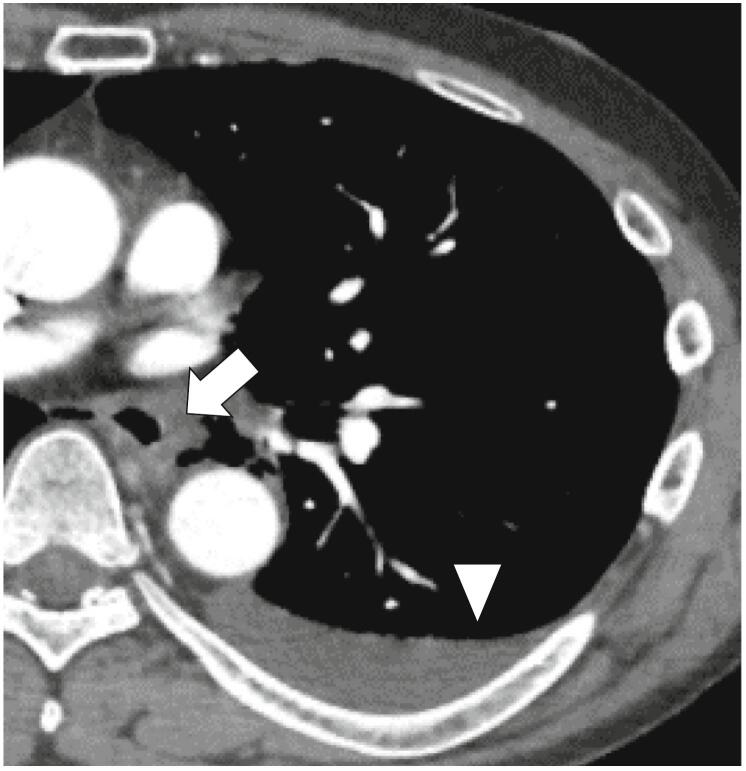
A chest CT showing the mediastinal fluid (arrow) and left pleural effusion (arrowhead).

Laboratory exams showed lactic acidosis (pH 7.159, lactate 13.7 mmol/L) and leukocytosis without a leftward shift. The Sequential Organ Failure Assessment (SOFA) score and quick SOFA score were 0 and 1, respectively, indicating the absence of sepsis or systemic inflammatory response syndrome. The electrocardiogram showed sinus tachycardia without any ST-segment changes. Enhanced Computed Tomography (CT) revealed mediastinal fluid with a left pleural effusion without active contrast extravasation (Fig. 1). Despite the absence of specific radiological and clinical signs, his clinical course raised suspicion of Boerhaave syndrome. He underwent emergency surgery 3 h and 30 min after the symptom onset at a tertiary care hospital. Meropenem was initiated prior to surgery and administered for three days at a dosage of 3 g per day, every 8 h. It was then switched to Sulbactam/Ampicillin for an additional three days, with a dosage of 9 g per day, divided every 12 h.

**Fig. 2:**
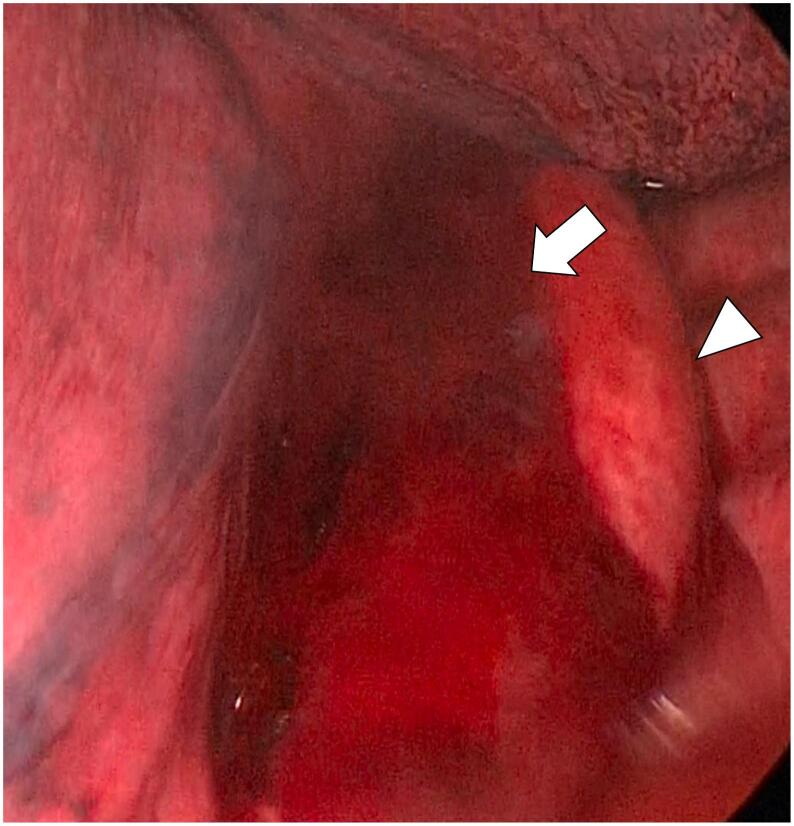
The posterior mediastinum was distended with a hematoma (arrow) along with the descending aorta (arrowhead). There was no food residue in the thoracic cavity.

**Fig. 3:**
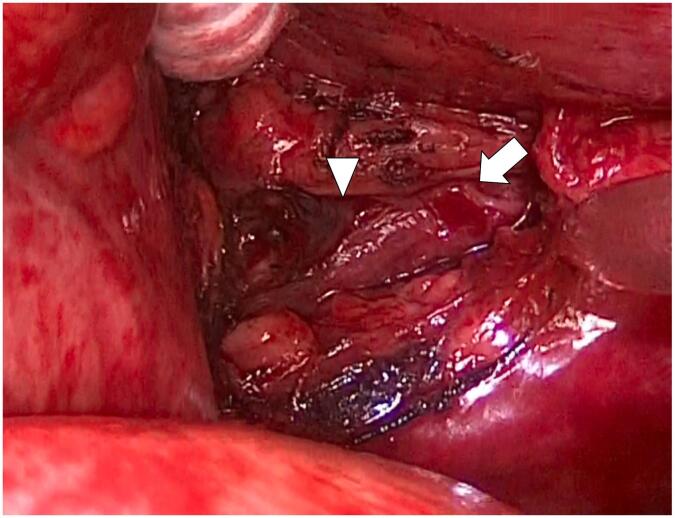
Active bleeding was seen coming from the proper esophageal artery (arrow) torn by the lacerated esophageal wall (arrowhead).

Thoracoscopic findings revealed a 280 ml hemorrhagic pleural effusion and a posterior mediastinal hematoma without food residue (Fig. 2). On thoracotomy, active bleeding occurred suddenly upon dividing the mediastinal pleura, and the esophageal content was released into the thoracic cavity simultaneously. The proper esophageal artery was found torn by the longitudinal wall laceration, resulting in a hematoma formation (Fig. 3). Hemostasis was achieved using a silk ligature, followed by a direct suture repair of the wall laceration with the pericardial fat pad coverage. The operative time was 143 min with an additional blood loss of 80 g. Enteral nutrition was administered via the nasogastric tube the day following surgery, and transitioned to oral intake on the seventh postoperative day. The patient was discharged uneventfully 12 days after the operation. On physical examination 2 weeks after discharge, he was afebrile and well nourished. He is currently disease free at 18 months after surgery.

## 3 Discussion

Most cases of Boerhaave syndrome manifest intrathoracic contamination by undigested food residue, as documented in the literature [13]. While hemothorax is a well-known finding in perforated peptic ulcers, including Boerhaave syndrome [14,15], the mediastinal hematoma observed in the present case may represent a rare form of impending esophageal perforation before intrathoracic bleeding occurs. This case highlights vital aspects. Firstly, a spontaneous esophageal perforation can manifest as a mediastinal hematoma due to the subpleural arterial injury caused by the esophageal wall laceration. This mediastinal bloody tamponade prevented food residue spillage until the division of the mediastinal pleura during the surgery in the present case. Therefore, a preoperative thoracentesis may have been inconclusive due to the absence of food residue, a conventional specific finding of BS, potentially leading to the treatment delay [11,13,16]. Conversely, those results also suggest that bloody thoracic drainage could serve as an alternative diagnostic sign of BS [17].

Secondly, a mediastinal hematoma may indicate a potentially fatal condition other than bacterial contamination in BS. While the clinical outcome of BS is mainly influenced by the level of intrathoracic food residue contamination [18], unexpected sudden bleeding could have resulted in a catastrophic outcome even before the food spillage became evident without a timely surgical intervention in the present case. These findings highlight the importance of a prompt surgical intervention as the mainstay treatment for BS, regardless of any intrathoracic manifestation. Although we performed thoracotomy in the present case, less invasive endoscopic surgery might be a feasible option [19,20]. The clinical and radiological findings were otherwise typical, emphasizing the significance of a comprehensive diagnostic assessment for BS.

## 4 Conclusion

BS can manifest as a mediastinal hematoma, and the absence of gastrointestinal content in the thoracic drainage does not rule out the possibility of BS. A mediastinal hematoma itself can result in a catastrophic outcome even before the food residue spillage becomes evident.

## List of abbreviations

BS: Boerhaave syndrome
SOFA: Sequential organ failure assessment
CT: Computed tomography

## Ethical approval

Not applicable.

## Funding

None.

## Author contribution

Yusuke Nakano wrote this paper. All authors read and approved the final manuscript. We thank Mr. John Martin for his proof-reading of the manuscript.

## Guarantor

Dr. Toru Nakamura.

## Research registration number

Not applicable.

## Consent for publication

Written informed consent was obtained from the patient for publication of this case report and accompanying images. A copy of the written consent is available for review by the Editor-in-Chief of this journal on request.

## Provenance and peer review

Not commissioned, externally peer-reviewed.

## Conflict of interest statement

The authors declare that they have no competing interests.

## Data Availability

Not applicable.
